# Data on a delivery of biomolecules into *Nicothiana benthamiana* leaves using different nanoparticles

**DOI:** 10.1016/j.dib.2017.12.031

**Published:** 2017-12-21

**Authors:** Antonida V. Makhotenko, Ekaterina A. Snigir, Natalia O. Kalinina, Valentin V. Makarov, Michael E. Taliansky

**Affiliations:** a DokaGene Ltd., Rogachevo, Moscow Region, Russia; b Lomonosov Moscow State University, Leninsky Gory, Moscow 119992, Russia; c James Hutton Institute, Invergowrie, DD2 5DA Dundee, UK

## Abstract

Nanoparticles (NPs) have a number of unique properties associated with their ultrasmall size and exhibit many advantages compared with existing plant biotechnology platforms for delivery of proteins, RNA and DNA of various sizes into the plant cells (Arruda et al., 2015; Silva et al., 2010; Martin-Ortigosa et al., 2014; Mitter et al., 2017) [1], [2], [3], [4]. The data presented in this article demonstrate a delivery of biomolecules into *Nicotiana benthamiana* plant leaves using various types of NPs including gold, iron oxide and chitosan NPs and methods of biolistic bombardment and infiltration. The data demonstrate physical characteristics of NPs coated with fluorescently labeled protein and small RNA (size and zeta-potential) and visualization of nanocomplexes delivery into cells of *N. benthamiana* leaves by fluorescence microscopy.

**Specifications Table**

**Table:** 

Subject area	*Biotechnology*
More specific subject area	*Plant biotechnology*
Type of data	*Table and images*
How data was acquired	*Dynamic laser light scattering (DLS) and zeta-potential data on characterization of NPs were obtained on Malvern Zetasizer instrument - NanoZS, Malvern Instruments, UK. Fluorescence microscope Axiovert 200* *M, Carl Zeiss,Germany was used for images of fluorescently labeled biomolecules in plant cells.*
Data format	*Analyzed data*
Experimental factors	*Fluorescence labeling of protein (bovine serum albumin with fluorescein isothiocyanate; BSA-FITC) and small RNA (tRNA with cyanine 3; tRNA-Cy3), assembly of nanoplatforms consisting of (gold, iron oxide and chitosan) NPs and fluorescently labeled biomolecules, delivery of nanocomplexes into N. benthamiana leaves by biolistic bombardment (for gold and iron oxide NPs) and syringe infiltration (for chitosan NPs)*
Experimental features	*Characterization of NPs coated with biomolecules (size and zeta potential), visualization of labeled biomolecules delivered into cells of N. benthamiana leaves by fluorescence microscopy*
Data source location	*DokaGene Ltd., Rogachevo, Moscow Region, Russia, and Belozersky Institute of Physico-Chemical Biology, Lomonosov Moscow State University, Leninsky Gory, Moscow, 119992, Russia*
Data accessibility	*Data are provided with this article*

**Value of the data**

• The data describe the assembly and characteristics of nanocomplexes consisting of different (gold, iron oxide and chitosan) NPs and fluorescently labeled protein (BSA-FITC) and small RNA (tRNA-Cy3).
• The data demonstrate delivery of the nanocomplexes into *N. benthamiana* leaves using two different techniques (biolistic bombardment and syringe infiltration) with subsequent visualization of fluorescently labeled biomolecules inside epidermis cells.
• The data may be used for development of easy and effective methods of biologically active component delivery to plant cells for different biotechnological applications.

## 1 Data

**Fig. 1:**
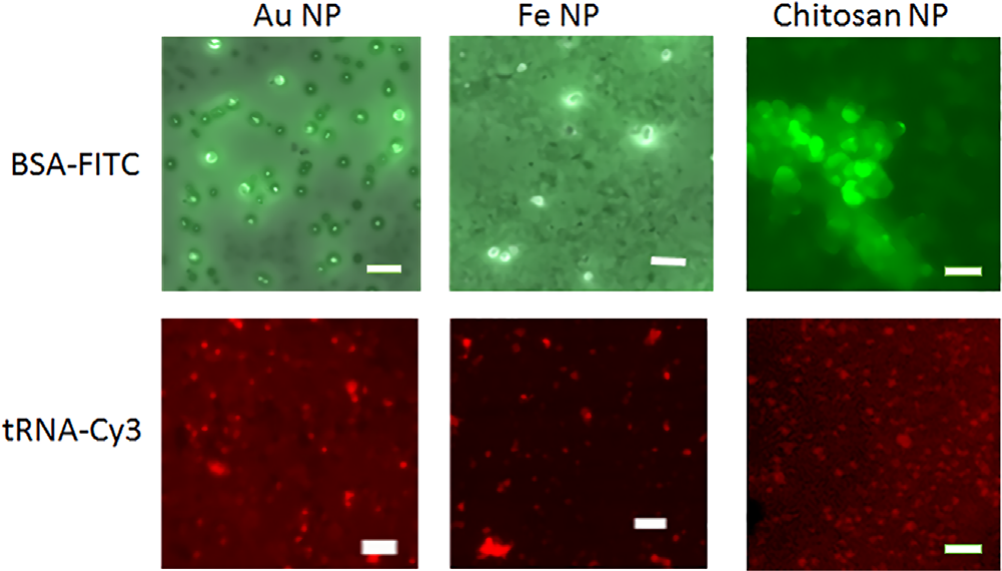
Fluorescence microscopy images of BSA-FITC and tRNA-Cy3 loaded onto gold (Au NP), iron oxide (Fe NP) and chitosan nanoparticles (Chitosan NP). White scale bar corresponds to 1 μm. To load NPs with protein, a mixture consisting of BSA-FITC (0.25 μg/ml), the buffer solution and a suspension of NPs (2 μl) was incubated at room temperature for 30 min under stirring. To load NPs with tRNA-Cy3, a suspension of NPs and RNA solution were mixed and incubated for 30 min at room temperature under stirring. Before further using, obtained nanocomplexes were sonicated.

**Fig. 2:**
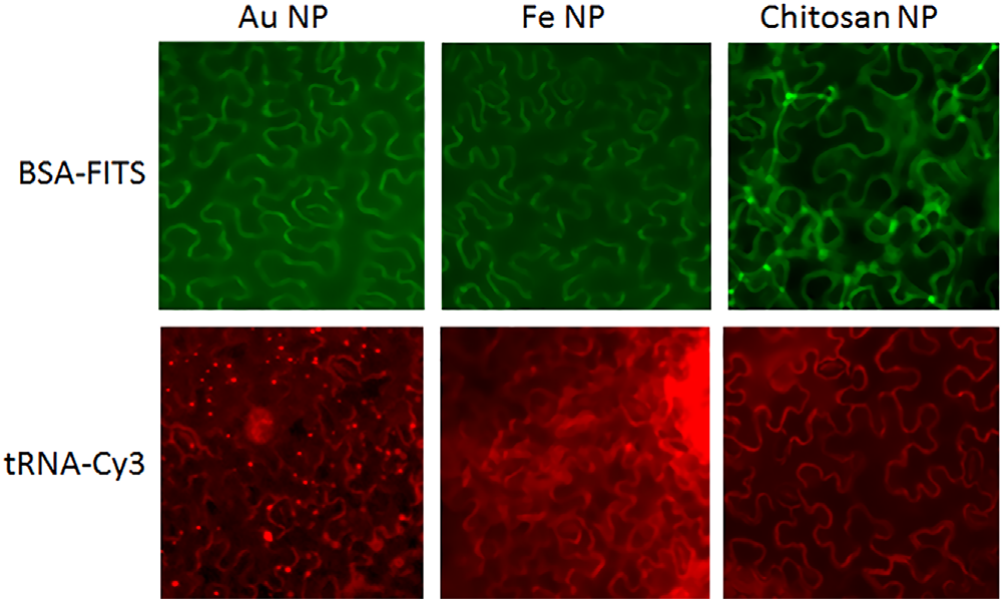
Fluorescence microscopy images of BSA-FITC and tRNA-Cy3 after their delivery into epidermal cells of *N.benthamiana* leaves using gold (Au NP), iron oxide (Fe NP) and chitosan nanoparticles (Chitosan NP) as delivery tools. Au NPs and Fe NPs were delivered into cells of *N. benthamiana* leaves by particle biolistic bombardment using PDS-1000/He gene gun (Bio-Rad). Chitosan nanocomplexes (after sonication) were infiltrated into leaves by syringe from the bottom side of the leaves.

The data presented demonstrate loading capacity of the fluorescently labeled protein (BSA-FITC) and small RNA (tRNA-Cy3) on the surface of the gold, iron oxide and chitosan NPs (Fig. 1) and represent physical characteristics of assembled nanocomplexes built on gold and iron oxide NPs (Table 1, DLS and zeta-potential data). In Fig. 2, the images of plant cells were taken after delivery of nanocomplexes containing the fluorescently labeled BSA and tRNA into *N. benthamiana* leaves. *N. benthamiana* has been chosen as a model experimental plant highly convenient for plant biology and microscopy studies [5].

**Table 1:** Physical characteristics of nanocomplexes (nanoparticles coated with biomolecules) by DLS method.

	**Au NP**		**Fe NP**	
	Size, (nm)	ζ-potencial(mV)	Size (nm)	ζ-potencial(mV)
BSA	712±95	−1.73±0.2	255±170	2.11±0.66
tRNA	955±5	−6.70±1.39	256±130	6.72±2.1

## 2 Experimental design, materials and methods

### 2.1 Nanocomplex assembly

We used commercial preparations of gold (1652262, Bio-Rad) and iron oxide (MP025-PEG-1ML, Nanocs) NPs. Chitosan NPs were prepared using tripolyphosphates (TPP), as described previously [6]. The labeling of BSA with FITC and total tRNA preparation with Cy3 were performed according to the standard protocols as described previously [7], [8]. The protein was loaded onto NPs in the buffer (250 мМ NaCl, 15 мМ Tris HCl, pH 8.0). A mixture consisting of BSA-FITC (0.25 μg/ml), the buffer and a suspension of NPs (2 μl) was incubated at room temperature for 30 min under stirring. To load NPs with tRNA-Cy3, a suspension of NPs and RNA solution were mixed and incubated for 30 min at room temperature under stirring. Before further using, obtained nanocomplexes were sonicated.

### 2.2 Nanocomplex characterisation

For DLS analysis assembled nanocomplexes (gold and iron NPs coated with BSA-FITC or tRNA-Cy3) were placed into the Zetasizer Nano ZS 1 cm cell of (Malvern Instruments, UK), and measurements were obtained using the He−Ne laser (633 nm). Curves were fitted using Dispersion Technology Software (DTS) version 5.10.

### 2.3 Nanocomplex delivery into *N. benthamiana* leaves

Nanocomplexes were delivered into cells of *N. benthamiana* leaves by particle biolistic bombardment using PDS-1000/He gene gun (Bio-Rad). Obtained nanocomplexes were spread directly onto the carrier and allowed to air-dry at room temperature for about 2 h. The following conditions: rupture disk pressure – 900 psi; target distance – 9 cm for gold NPs and 6 cm for iron oxide NPs. were used as a standard bombardment protocol for *N. benthamiana* leaves. Chitosan nanocomplexes after sonication were infiltrated into leaves by syringe from the bottom side of the leaves. Bright-field and fluorescence images were acquired 2 days after delivery using a Axiovert 200 M microscope (Carl Zeiss) equipped with an Plan-Neofluar 10× and 20× objectives. Pictures were taken using ORCA II ERG-2 digital camera (Hamamatsu Photonics, Japan) and AxioVision LE software.
